# Early Detection of Chronic Kidney Disease Using Plasma Neutrophil Gelatinase-Associated Lipocalin and Kidney Injury Molecule-1 in Small-Breed Dogs: A Retrospective Pilot Study

**DOI:** 10.3390/ani14162313

**Published:** 2024-08-09

**Authors:** Hyo-Sung Kim, Han-Jun Kim, Sun-Hee Do

**Affiliations:** 1 Department of Veterinary Clinical Pathology, College of Veterinary Medicine, Konkuk University, Seoul 05029, Republic of Korea; 2 Department of Veterinary Clinical Pathology, Konkuk University Animal Medical Center, Konkuk University, Seoul 05029, Republic of Korea; 3 College of Pharmacy, Korea University, Sejong 30019, Republic of Korea

**Keywords:** canine, biomarkers, serum creatinine, symmetrical dimethylarginine, International Renal Interest Society, staging system, early diagnosis

## Abstract

**Simple Summary:**

Early detection of kidney diseases in dogs is crucial. While many studies focus on urine biomarkers, this study explored the potential of plasma biomarkers. We investigated plasma neutrophil gelatinase-associated lipocalin (pNGAL) and plasma kidney injury molecule-1 (pKIM-1) in small-breed dogs for detecting chronic kidney disease (CKD). Our findings suggest that pNGAL and pKIM-1 can identify CKD stages and risk groups effectively. These biomarkers showed better diagnostic accuracy than traditional indicators like serum creatinine. Therefore, pNGAL and pKIM-1 could be valuable tools for early CKD detection in veterinary practice.

**Abstract:**

Multiple diagnostic modalities are urgently needed to identify early-stage kidney diseases. Various molecules have been investigated; however, most studies have focused on identifying specific biomarkers in urine. Considering that assessing the symmetrical dimethylarginine (SDMA) plasma concentration is more suitable as an early diagnostic test for chronic kidney disease (CKD) in routine veterinary practice, we aimed to investigate the clinical usefulness of plasma neutrophil gelatinase-associated lipocalin (pNGAL) and plasma kidney injury molecule-1 (pKIM-1) concentrations for CKD detection in small-breed dogs. Through a retrospective analysis, we found that numerous clinicopathological data showed a log-normal distribution, even when they satisfied normality tests. Moreover, the log-transformed pNGAL and pKIM-1 concentrations successfully identified CKD International Renal Interest Society (IRIS) stages 1–4 and the risk group with underlying CKD risk factors. Correlation analysis and group comparison of other factors confirmed the possibility of using these two biomarkers for detecting the CKD risk group and IRIS stage 1. Receiver operating characteristic curve analysis revealed that the diagnostic accuracy for discriminating the risk group was superior in the order of pKIM-1, pNGAL, SDMA, and serum creatinine levels. In conclusion, these results suggest that pKIM-1 and pNGAL are possible early or quantifiable markers of insignificant CKD or can be at least used as an adjunct with traditional indicators.

## 1. Introduction

Chronic kidney disease (CKD) can induce severe consequences in most affected animals, as it progressively worsens if undetected and untreated; therefore, early diagnosis of CKD is required for effective management [1]. Moreover, severe kidney disease may create fluid overload and reduce clearance in veterinary clinics, where the application of hemodialysis is challenging [2]. Therefore, diagnostic techniques are essential for monitoring kidney function, detecting the early phase of CKD, and enabling the timely implementation of preventive measures and intervention strategies. Distinguishing individuals in the early stage of CKD from healthy individuals allows clinicians to initiate targeted monitoring and early interventions, including dietary modifications, and reno-protective therapies. This proactive approach can effectively slow or halt disease progression and prevent the development of complications [3].

Conducting renal biopsy and calculating the glomerular filtration rate (GFR) are the current gold standard methods for determining the type of disease (such as tubular and ischemic injuries) and renal function in humans and animals; however, their clinical use has been limited owing to impracticality [4,5,6,7,8,9,10]. Instead, the estimated GFR, which is calculated using serum creatinine (sCr) and/or cystatin C concentrations and considering other factors such as race, sex, age, and body surface area, is frequently used in human medicine [11]. However, such an estimation system has not been applied in veterinary medicine. Indirect methods of estimating GFR are in routine use and mostly include blood urea nitrogen (BUN), sCr, and the recently developed symmetrical dimethylarginine (SDMA) [12,13]. Unfortunately, sCr is a nonlinear marker of GFR, and has low specificity influenced by muscle mass, hydration status, and the analysis method [7,14,15,16,17]. SDMA has been in the spotlight as a GFR surrogate marker because, in some circumstances, SDMA offers an advantage over sCr as it is unaffected by muscle mass [3,18,19]. However, like sCr, SDMA has limitations since it may require a longitudinal evaluation and does not reflect tubular damage [14]. Tubular injury, the principal mechanism of acute kidney injury (AKI), can precede GFR reduction in humans and animals [20,21,22]. Even after GFR alteration, sCr shows only a small increase [20]. Similarly, SDMA does not respond immediately after renal insult [23]. Urinalysis, including urine-specific gravity, is currently used to detect a decrease in urine concentrating ability and tubular injury; however, it lacks sensitivity or specificity [14]. Therefore, a renal index that can diagnose kidney damage earlier and reflect tubular damage more effectively than existing GFR markers is needed.

An ideal biomarker should be inexpensive, accurate, and capable of diagnosing disease severity and prognosis even in the early stages [24]. However, no single marker perfectly satisfies all criteria, and research on multiple diagnostics is required to overcome this limitation [14]. Numerous studies have been conducted to develop markers of early kidney injury, and several proteins have been approved by the Food and Drug Administration, European Medicines Agency, and Pharmaceuticals and Medical Devices Agency [1,25,26]. Neutrophil gelatinase-associated lipocalin (NGAL) and kidney injury molecule-1 (KIM-1) are among the better studied prospective markers of AKI and CKD in veterinary and human medicine [1].

As the name indicates, NGAL is expressed by neutrophils and several other cells, including renal tubular epithelial cells [27]. The kidneys rapidly clear plasma NGAL, with an estimated half-life of 10–20 min [28]. Plasma NGAL is excreted via glomerular filtration and undergoes complete reabsorption in healthy tubular cells [29]. The ascending tubules of the loop of Henle and collecting tubules rapidly and vastly express NGAL in response to renal epithelial injury [24,27,30]. Due to the reabsorption of plasma NGAL in the proximal tubules, elevated concentrations can result either from disrupted NGAL reabsorption or increased NGAL synthesis caused by damage to the proximal tubules [24]. Consequently, NGAL has been studied as a real-time indicator of kidney damage in human medicine; however, its clinical application is still being established [31,32]. Furthermore, NGAL levels correlate with kidney damage severity in patients with CKD [24].

KIM-1, also known as T-cell immunoglobulin mucin domain-1 owing to its low expression in activated T-cells, is expressed in necrotic and regenerating proximal tubules in humans and animals [33,34,35,36,37]. Following an injury to the proximal tubules, an excess of KIM-1 protein may be released not only into the urine but also into the bloodstream [33]. In addition to acute injury, elevated KIM-1 expression under hypoxic conditions promotes CKD progression, creating a positive feedback loop [38]. Therefore, KIM-1 has drawn attention as an emerging biomarker in humans and animals, and its role in chronic diseases has been proposed [22,35,38].

NGAL and KIM-1 have been suggested as biomarkers of early kidney disease in human and veterinary medicine, as both represent tubular injury [24,39]. Similar to several other biomarkers, research has focused on urinary NGAL and KIM-1 concentrations for clinical evaluation in human and veterinary medicine [14,40]. To address the knowledge gap in research on diagnostic biomarkers, which currently focuses on urine, we conducted the present retrospective study, which aimed to confirm the utility of NGAL and KIM-1 as plasma markers for the early detection of CKD. This study analyzed NGAL and KIM-1 concentrations in dog blood samples and compared them with those of serum urea nitrogen, creatinine, and SDMA, which are widely used in veterinary hospitals. We hypothesized that plasma NGAL (pNGAL) and plasma KIM-1 (pKIM-1) concentrations may differ between the control group and the group with risk factors for CKD and between the International Renal Interest Society (IRIS) CKD stage 1 and stage 2–4 groups.

## 2. Materials and Methods

### 2.1. Study Population

This retrospective study was conducted using archived plasma samples and medical records of dogs admitted to the Konkuk University Veterinary Medical Teaching Hospital from 1 July 2018 to 31 August 2022. Ethical approval was not required for this animal study owing to its retrospective nature. During this period, only small dogs weighing <10 kg were selected for testing to minimize the effect of body weight [15,41]. Additionally, cases with insufficient remnant volumes or hemolysis were excluded because they may affect the enzyme-linked immunosorbent assay results. Finally, to minimize bias, only samples with measured serum SDMA, BUN, and creatinine levels were selected for the population in this study. Dogs with no major abnormalities in clinical symptoms, clinical pathology tests, or radiologic examination, no long-term medication use or underlying diseases, and those who visited the hospital for general examinations, such as mild patellar luxation or cranial cruciate ligament rupture, were used as the control group. The test group comprised dogs diagnosed with CKD or those identified as having risk factors for CKD. Blood samples from these dogs were collected prior to anesthesia or any other veterinary procedures.

### 2.2. Sample Processing and Biomarker Analysis

The collected venous blood was processed separately in ethylenediaminetetraacetic acid (EDTA)-containing tubes for complete blood count (CBC) analysis and in lithium heparin tubes for serum chemistry analysis. The CBC was measured using a Procyte Dx hematology analyzer (IDEXX Laboratories, Westbrook, ME, USA). Heparinized whole blood was centrifuged at 3000 rpm for 6 min at room temperature, and serum chemistry was measured using a Catalyst One chemistry analyzer (IDEXX Laboratories). Urine protein and creatinine levels were measured in fresh urine samples using the Catalyst One chemistry analyzer, and urine-specific gravity was measured using the refractometer (Richert, Buffalo, NY, USA). After clinical use, the EDTA whole blood remnant was separated by centrifugation at 3000 rpm for 6 min at room temperature. Residual EDTA or heparinized plasma samples were then frozen for 6 h at −20 °C and subsequently transferred to a storage temperature of −78 °C for up to 1 year until batched for pNGAL and pKIM-1 analysis.

pNGAL and pKIM-1 concentrations in condition-satisfied dogs were analyzed using frozen plasma samples. The pNGAL and pKIM-1 concentrations were determined using a canine-specific sandwich enzyme-linked immunosorbent assay kit (ab205085 and ab205084, respectively; Abcam, Cambridge, UK), following the manufacturer’s instructions. The intra- and inter-coefficients of variability were below 10% for both kits. The frozen plasma samples were rapidly thawed in a water bath at 37 °C. Subsequently, the plasma was diluted to 1:10 for pKIM-1 and 1:50 for pNGAL using the sample diluent contained within the assay kit. Absorbance was measured at 450 nm using a microplate reader (Tecan, Zurich, Switzerland) following the manufacturer’s instructions. All samples were analyzed in duplicate.

### 2.3. Chronic Kidney Disease Staging and Group Classification

The study population was divided into groups based on the CKD stage according to the 2023 IRIS staging system [42]. Owing to the retrospective nature of this study, only the sCr and SDMA concentrations on the collection date of the evaluated plasma were used for staging. However, due to the limited availability of urinalysis information in the medical records and the need to ensure a sufficient sample size for each group, sub-staging based on blood pressure measured using a sphygmomanometer (Cardiac Direct, Ventura, CA, USA) and urinalysis was not implemented. Dogs in which abnormalities in renal structure, uroliths and renal cysts, were radiologically confirmed among subjects with sCr concentrations < 1.4 mg/dL and SDMA concentrations < 18 µg/dL were categorized as having CKD stage 1. Furthermore, those with only risk factors, such as myxomatous mitral valve disease (MMVD) (n = 5), a portosystemic shunt (n = 1), chronic heart failure (n = 2), hyperadrenocorticism (HAC) (n = 4), diabetes mellitus (n = 1), and babesiosis (n = 1), were classified as the risk group [43]. However, no kidney abnormalities were observed in subjects in the risk group according to radiologic examination. Subjects with sCr concentrations of 1.4–2.8 mg/dL or SDMA concentrations of 18–35 µg/dL were classified as having CKD stage 2. Subjects with sCr concentrations of 2.9–5.0 mg/dL or SDMA concentrations of 36–54 µg/dL were classified as having CKD stage 3. Subjects with sCr concentrations >5.0 mg/dL or SDMA concentrations > 54 µg/dL were classified as having CKD stage 4. No dog exclusively presented with prerenal or postrenal azotemia among those with CKD stages 1–4. Stage 4 subjects were unified into the stage 3–4 group, as only three dogs were in stage 4.

### 2.4. Statistical Analysis

Statistical analyses and visualization were performed using GraphPad Prism version 9.3.1 (GraphPad Software, San Diego, CA, USA) and SPSS version 20 (IBM Corp., Armonk, NY, USA). Categorical variables, such as sex and body condition scores, were analyzed using the chi-squared test. Differences in normally distributed datasets among the five groups, including log-transformed data, were analyzed using a one-way analysis of variance followed by the Bonferroni post hoc test. Age on the day of sample collection was converted to decimal values and regarded as a continuous variable. The relationship between parametric measurements was determined using Pearson’s bivariate correlation coefficient. Point-biserial correlations were used to investigate the relationship between biomarkers and concurrent diseases. Pairwise comparisons of correlation coefficients between kidney biomarkers were conducted using the Z-test after Fisher transformation. The optimal cut-off values of the four kidney biomarkers were determined using receiver operating characteristic (ROC) curves, selecting the optimal cut-off value using the concordance probability method and the highest value for the formula: sensitivity × specificity value [44]. All statistical analyses were two-tailed tests of statistical significance. Statistical significance was set at *p* < 0.05.

## 3. Results

### 3.1. Relationship and Distribution of Each Plasma Kidney Injury Biomarker

**Figure 1:**
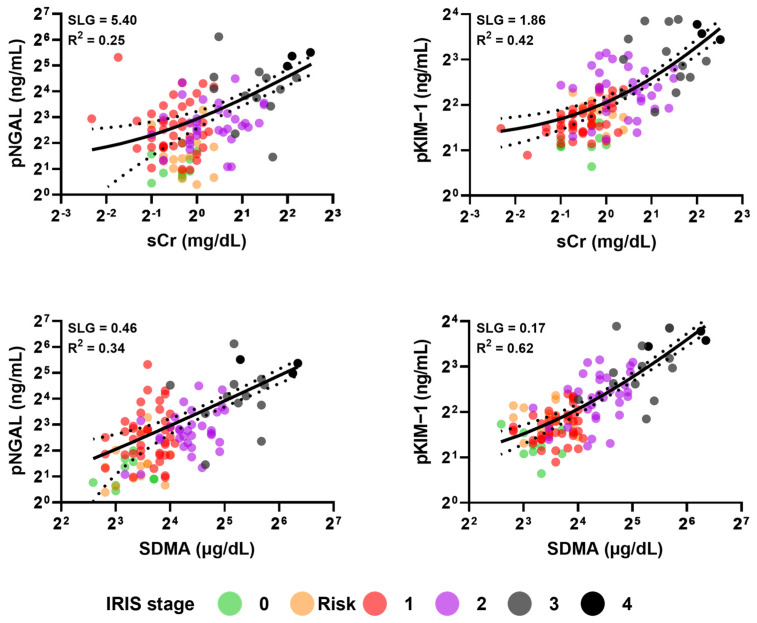
Correlation between traditional and novel kidney injury biomarkers. Biomarkers showed a linear relationship with each other after the X and Y axes were displayed as log scales. Each scattered dot represents an individual case, colored by the chronic kidney disease stage. The line indicates simple linear regression, accompanied by small black dots describing 95% confidence intervals. Abbreviations: SLG, slope gradient; R^2^, coefficient of determination (R-squared), indicating the goodness-of-fit measure of the linear model; pNGAL, plasma neutrophil gelatinase-associated lipocalin; sCr, serum creatinine; SDMA, symmetrical dimethylarginine; IRIS, International Renal Interest Society.

**Figure 2:**
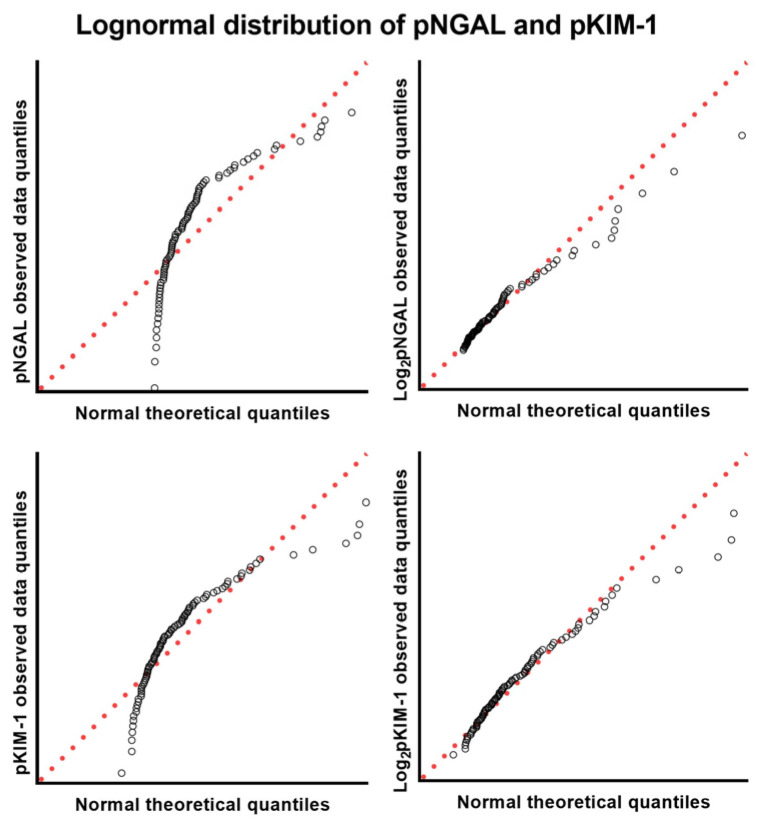
Normality and log-normality analysis of novel kidney injury biomarkers. pNGAL and pKIM-1 showed a log-normal distribution. Q-Q plots of two objective biomarkers were compared before and after logarithmic transformation. Q-Q plots of all other data are presented in Supplementary Figure S2. Red dotted lines indicate the identity line, whereas points forming a straight line indicate a suitable distribution. Abbreviations: pNGAL, plasma neutrophil gelatinase-associated lipocalin; pKIM-1, plasma kidney injury molecule-1.

A total of 117 plasma samples were collected, and based on the IRIS guidelines, 11, 14, 43, 33, and 16 dogs were classified into the control, risk, IRIS stage 1, IRIS stage 2, and IRIS stage 3–4 groups, respectively. After grouping, the relationships between the new biomarkers (i.e., pNGAL and pKIM-1) and existing biomarkers (i.e., sCr and SDMA) were analyzed. When the four concentration pairs were plotted as scatter plots, there were fewer overlapping points for SDMA and pKIM-1 than for the other pairs (Figure 1, plot with log-transformed axes). This phenomenon became more apparent when the axis was expressed as an exponential power of two instead of a linear power (Supplementary Figure S1, plot with linear axes). Moreover, pNGAL and pKIM-1 showed a linear relationship with sCr and SDMA. The coefficient of determination (R^2^), which indicates how close the data distribution approaches the linear regression model, did not exceed 0.65 in any comparison, suggesting that the model could not explain more than 35% of all cases. However, the SDMA-pKIM-1 relationship was the closest to the linear model, and the distance between pNGAL and sCr was the farthest. The four biomarkers showed exponential characteristics; hence, the normality and log-normality of the values were confirmed using a quantile–quantile plot before statistical analysis. All four biomarkers satisfied log-normality even if they satisfied both normality and log-normality tests (Figure 2, Supplementary Figure S2).

### 3.2. Identification of the Risk and Stage 1 Groups Using log_2_pKIM-1 and log_2_pNGAL Markers

**Figure 3:**
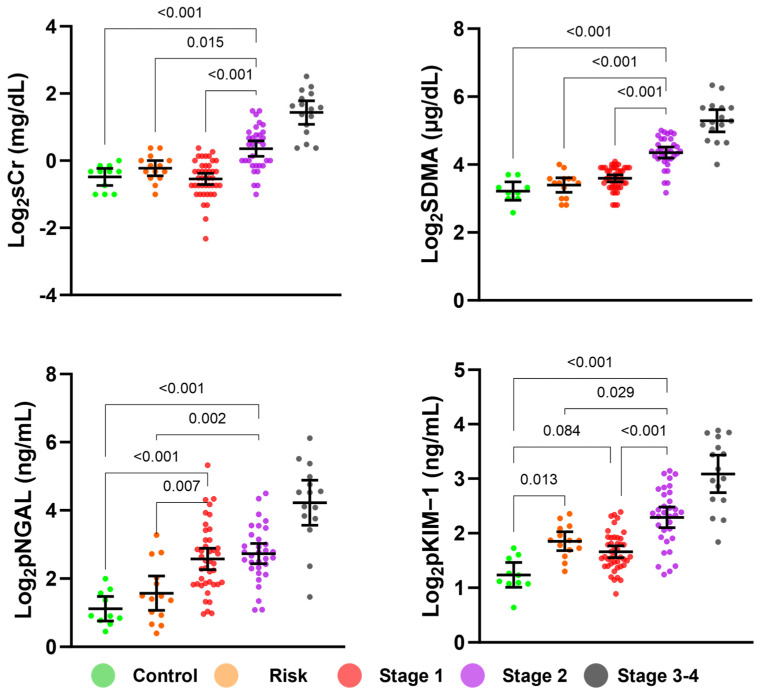
Statistical analysis of differences in the concentrations of biomarkers between the groups after log transformation. No significant differences in serum creatinine (sCr) and symmetrical dimethylarginine (SDMA) concentrations were observed between the control, risk, and stage 1 groups, whereas a significant difference in pNGAL and pKIM-1 was found. The *p*-value between the stage 3–4 group and other groups was omitted because they were all <0.001 for four biomarkers. The lines indicate a mean ± 95% confidence interval.

According to the data distribution, a suitable statistical method was identified. After converting the data to a base 2 logarithm, differences in renal biomarker concentrations according to the CKD stage were analyzed (Figure 3). Regardless of the logarithmic transformation, the four biomarkers significantly differed between the stage 3–4 group and other groups (Figure 3, Supplementary Figure S3). Likewise, when converted to logarithms, the four markers were significantly different between the stage 2 group and other groups, except for log_2_pNGAL, which was comparable between the stage 1 and stage 2 groups. sCr and SDMA levels are the criteria for classifying the CKD IRIS stages; however, the control, risk, and stage 1 groups were not distinguished by Log_2_sCr and log_2_SDMA. Interestingly, log_2_pKIM-1 exhibited statistically significant differences between the control and risk groups and between the stage 1 and stage 2 groups. In contrast, log_2_pNGAL differentiated between the control and stage 1 groups and the risk and stage 1 groups.

### 3.3. Correlation between Biomarkers and Other Clinical Factors

**Figure 4:**
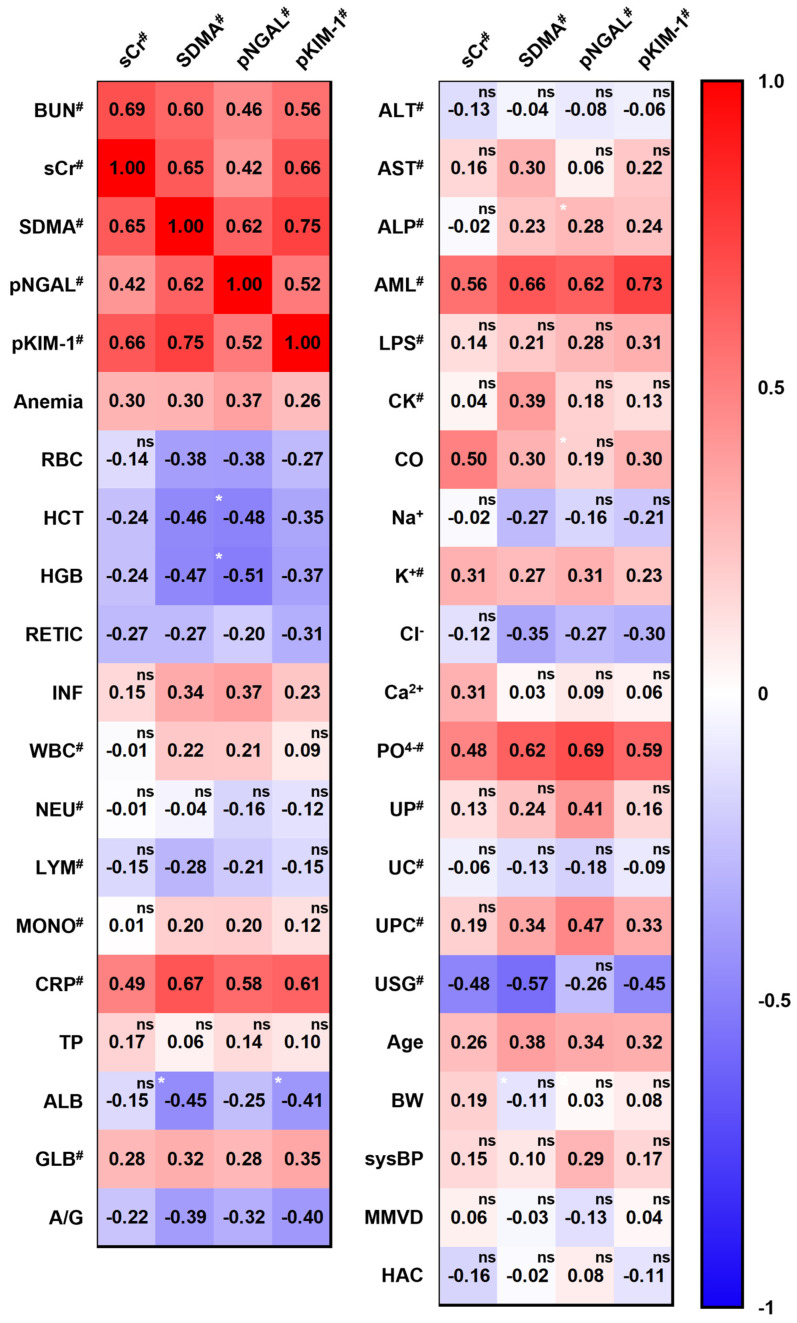
Correlation matrix between kidney biomarkers and clinicopathological indicators. The numbers in each cross box are r coefficients, indicating linearity. Red and blue correspond to positive and negative correlations, respectively. Hashes (#) indicate that the markers were analyzed after log_2_ transformation, as they showed a log-normal distribution. The black superscripted “ns” indicates that the correlation was not statistically significant; otherwise, the *p*-value was <0.05. A white superscripted asterisk (*) indicates a comparison with sCr; *, *p* < 0.05. Abbreviations: BUN, blood urea nitrogen; sCr, serum creatinine; SDMA, symmetric dimethylarginine; pNGAL, plasma neutrophil gelatinase-associated lipocalin; pKIM-1, plasma kidney injury molecule-1; RBC, red blood cell; HCT, hematocrit; HGB, hemoglobin; RETIC, reticulocyte count; INF, inflammation; WBC, white blood cell; NEU, neutrophil count; LYM, lymphocyte count; MONO, monocyte count; CRP, C-reactive protein; TP, total protein; ALB, albumin; GLB, globulin; A/G, albumin-to-globulin ratio; ALT, alanine aminotransferase; AST, aspartate aminotransferase; ALP, alkaline phosphatase; AML, amylase; LPS, lipase; CK, creatinine kinase; CO, calculated osmolality; UP, urine protein concentration; UC, urine creatinine concentration; UPC, urine protein-to-urine creatinine ratio; USG, urine-specific gravity; BW, body weight; sysBP, systolic blood pressure; MMVD, myxomatous mitral valve disease; HAC, hyperadrenocorticism.

All other data distributions were first reanalyzed to determine whether pNGAL and pKIM-1 levels increased due to other factors, such as disease states, age, and body weight. The majority of tests followed a log-normal distribution, including the white blood cell (WBC) count, urinalysis (urine protein, urine creatinine, urine protein creatinine ratio, urine-specific gravity), C-reactive protein (CRP), globulin, enzymes (alanine aminotransferase, aspartate aminotransferase, alkaline phosphatase, amylase, lipase, creatinine kinase), and electrolytes K^+^ and PO_4_^−^ levels (Supplementary Figure S2). Subsequently, the relationships between each biomarker, clinicopathological factors, and concurrent diseases were analyzed. Briefly, none of the other CBC or blood chemistry values showed a high correlation (r > 0.7) with the four biomarkers (Figure 4). The correlation coefficients for PO_4_^−^ and amylase concentrations, which are elevated in renal failure, reached moderate correlation coefficients. Additionally, the four objective biomarkers—namely, pNGAL, pKIM-1, sCr, and SDMA—exhibited positive, moderate (0.5–0.7) correlations with CRP. However, all other tests, including those for anemia-related CBC, inflammation-related CBC, albumin, globulin, lipase, creatine kinase, electrolytes, urine protein, urine creatinine, urine protein creatinine ratio, and urine-specific gravity, showed low (0.3–0.5) to negligible (<0.3) correlations.

In addition to the laboratory test results, we analyzed the correlation between the four kidney biomarkers and categorical variables, such as signalments and concurrent disease states (Figure 4). Age showed a low correlation, whereas body weight and systolic blood pressure revealed negligible correlations with all four biomarkers. The coefficient values of pNGAL for correlation with anemia and SDMA for inflammation were higher than those of the other kidney biomarkers; however, all four markers exhibited low correlations with anemia and inflammation. Additionally, MMVD (n = 57) and HAC (n = 41), the most commonly observed comorbidities in this population, did not show a significant correlation with kidney biomarkers, as demonstrated in a previous study showing that SDMA was not affected by any MMVD stage [45].

In addition to assessing individual correlations, we statistically compared correlation magnitudes between different biomarkers to identify any significant differences. The correlation coefficients for the associations of pNGAL-to-hemoglobin, pNGAL-to-hematocrit, SDMA-to-albumin, pKIM-1-to-albumin, and pNGAL-to-ALP were found to be significantly different from those of the sCr-to-each factor (Figure 4). However, no significant correlation differences were observed among other biomarkers.

### 3.4. Confirmation of Equivalence between Groups

After confirming that the correlations with clinicopathological indicators were not different between SDMA and the novel markers pNGAL and pKIM-1, we aimed to investigate whether the observed statistical differences between the groups could be attributed to differences in the study population. Overall, we confirmed that the differences between the control, risk, and stage 1 groups were not significant in any dataset or were at least within the normal range. The ages of dogs in the CKD groups were significantly higher than those in the control group; however, no significant differences in breed, sex, body weight, body condition score, or systolic blood pressure were observed among the groups (Table 1).

All median CBC, serum chemistry, and urinalysis results were within the reference intervals in the control, risk, and CKD IRIS stage 1 groups (Table 2 and Table 3). Several subjects in the three groups had an anemic and/or inflammatory status; however, no significant differences were identified in any factor among the three groups, except for ionized calcium levels. Additionally, the CRP, albumin-to-globulin ratio, and sodium levels were the only markers that showed statistically significant differences between the IRIS stage 2 and pre-stage groups. Only the IRIS stage 3–4 groups showed significant differences in clinical pathology test results, as compared with the other groups.

### 3.5. Clinical Efficacy Comparison and Gray Area Determination of Renal Biomarkers

**Figure 5:**
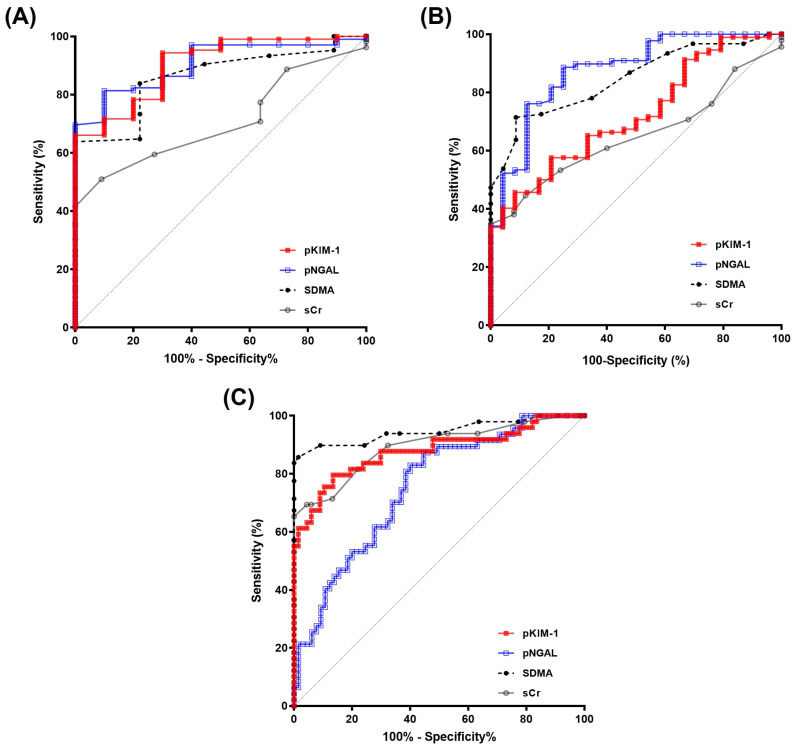
Accuracy of the biomarkers in detecting chronic kidney disease. (**A**) The control group was classified as the negative instance, whereas the risk and IRIS stage 1–4 groups were classified as positive instances. Overall, the areas under the curves of pNGAL and pKIM-1 were found to be comparable and were slightly higher than those of SDMA; sCr exhibited the lowest AUC. (**B**) The control and risk groups were classified as negative instances, whereas the IRIS stage 1–4 groups were classified as positive instances. Overall, the areas under the curves were highest in the order of pNGAL, symmetrical dimethylarginine (SDMA), pKIM-1, and serum creatinine (sCr). (**C**) The control, risk, and IRIS stage 1 groups were classified as negative instances, whereas the IRIS stage 2–4 groups were classified as positive instances. Overall, the areas under the curves of pKIM-1 were higher than those of pNGAL. Abbreviations: AUC, area under the curve; pNGAL, plasma neutrophil gelatinase-associated lipocalin; pKIM-1, plasma kidney injury molecule-1.

After confirming that the differences in pNGAL and pKIM-1 levels between the groups were not influenced by bias in Section 3.3 and Section 3.4, the ROC curves were analyzed as a quantitative tool independent of group prevalence, sample selection, and test units [46]. First, the risk-distinguishing efficacy of the biomarkers was calculated by including the risk group in the positive test results (Figure 5A). Overall, test accuracy (area under the curve [AUC]), sensitivity, and specificity were similar between pKIM-1 and pNGAL, and they were higher than those of SDMA and sCr, with almost no overlap, except for one SDMA cut-off value point (10.50 µg/dL). Consistent with our previous results, the pNGAL and pKIM-1 AUC values showed high accuracy in discriminating the CKD risk group from the control group (AUC > 0.9), whereas the other two markers showed moderate accuracy (AUC > 0.7) (Table 4). Even at low sensitivity, pKIM-1 showed 100% specificity at the optimal cut-off value. Therefore, the positive likelihood ratio (LR^+^) of pKIM-1 was defined as having high clinical practicability to the extent that a decisive probability shift occurred when the test result was positive (LR^+^ > 10). The negative likelihood ratio (LR^−^) of SDMA, pNGAL, and pKIM-1 made small but sometimes significant changes in disease-free probability when the result was negative (0.2 < LR^−^ < 0.5).

An additional ROC curve analysis excluding the risk group for determining the CKD-positive status (Figure 5B,C, Table 5 and Table 6) was performed to ascertain the stage classifying ranges of each biomarker. When positive incidence was defined as greater than or equal to stage 1 and negative incidence was defined as control and risk, the diagnostic accuracy of pNGAL was comparable to that of SDMA, whereas the diagnostic accuracy of pKIM-1 was comparable to that of sCr (Figure 5B, Table 5). The diagnostic accuracy was the highest for pNGAL, followed by SDMA, pKIM-1, and sCr. Additionally, the optimal cut-off value of SDMA was similar to the normal limit of 14 mg/dL provided by IDEXX Laboratories. When positive incidence was defined as stage 2 or higher, the accuracy of SDMA and sCr tests was higher than that of other biomarkers because sCr and SDMA were criteria for IRIS stages (Figure 5C, Table 6). The ROC curve analysis indicated that the CKD risk index area was 0.95–1.4 mg/dL, 10.50–18 µg/dL, 3.30–4.20 ng/mL, and 3.33–4.14 ng/mL for sCr, SDMA, pNGAL, and pKIM-1, respectively.

## 4. Discussion

To date, no single renal biomarker has provided sufficient information regarding kidney disease, and an array system using multiple markers is urgently needed to maximize diagnostic sensitivity and specificity in the field of nephrology [39,47]. Therefore, many candidate substances, such as NGAL, KIM-1, and cystatin C, have been suggested as markers of the early stage of acute kidney injury and chronic kidney disease, and their pathological mechanisms have been studied [48]. However, their clinical usefulness is not well established, and they are mainly analyzed using urine samples [48]. We hypothesized that plasma concentrations of NGAL and KIM-1 could also be used as early diagnostic markers of CKD in small-breed dogs.

In the log-transformed data, sCr and SDMA could not discriminate between the control, risk, and stage 1 groups; however, pNGAL and pKIM-1 could distinguish significantly. These results suggest that pNGAL and pKIM-1 can be considered early biomarkers for identifying those at risk of CKD and radiologically confirmed stage 1 CKD in small-breed dogs, which is indistinguishable by conventional criteria. Moreover, the correlation between these two markers and the other test results did not differ from those of existing indicators, and group differences were minimal. The correlation analysis indicates that in the present study involving small dogs, the influence of the 41 factors on pNGAL and pKIM-1 is no greater than their influence on sCr or SDMA. The analysis of the study population indicates equivalence between the control, risk, and radiologically confirmed stage 1 groups in general clinical pathology tests and supports the efficacy of pNGAL and pKIM-1 for detecting the risk group for CKD. Finally, ROC curve analysis was used to calculate the optimal cut-off value and diagnostic accuracy for the risk group. Because the evaluation criterion was the sCr- and SDMA-based IRIS staging system, pNGAL and pKIM-1 were not superior to sCr and SDMA in differentiating stages 2–4 from stage 1 or the control group. However, pNGAL and pKIM-1 were more accurate than sCr or SDMA in differentiating the CKD risk group, and pNGAL was compatible with SDMA for stage 1–4 diagnosis. Therefore, we conclude that pNGAL and pKIM-1 concentrations can be used as markers for identifying CKD risk and stage 1 CKD or complementary markers to sCr and SDMA, as well as to establish novel diagnostic criteria and develop comprehensive indicators.

Both pNGAL and pKIM-1 can be influenced by the GFR. A cohort study involving human patients with CKD reported a moderate correlation between plasma KIM-1 levels and estimated GFR (eGFR) [49]. Similarly, our findings indicate that pKIM-1 displays a moderate correlation with SDMA and sCr, which are markers of GFR. However, another study observed no correlation between pNGAL and eGFR, contrasting our findings of a moderate correlation [50]. Notably, studies involving hypertensive and uremic patients have reported correlations between pNGAL and GFR, suggesting that variations in the study population contribute to these dissimilar outcomes [51,52]. While the urinary KIM-1 concentration reflects acute tubular damage, plasma KIM-1 concentration is believed to represent the cumulative effect of injury over time and its continuous production [49]. Although there are conflicting opinions regarding the relationship of pNGAL and pKIM-1 with GFR, previous studies have consistently supported that both pNGAL and pKIM-1 possess greater diagnostic value for CKD early detection than sCr.

Urinary NGAL is a novel biomarker for various kidney injuries, such as gentamicin-induced tubular damage [53,54,55,56], tenofovir disoproxil fumarate (an anti-human immunodeficiency virus agent)-induced tubular damage [57], heatstroke-induced kidney injury [58], envenomation-derived tubular injury [23], ischemia/reperfusion injury [59], X-linked hereditary nephropathy [60], and post-surgery kidney injury [61]. Moreover, a progressive increase in urinary NGAL levels has been shown to help distinguish CKD stages and diagnose mild kidney injury [10,62,63]. However, the clinical value of pNGAL is controversial. Similar to the pNGAL patterns observed in the present study, serum NGAL correlated with sCr and BUN and was reported to increase with the CKD stage or azotemia, suggesting its potential as an indicator of CKD severity [64,65]. Additionally, other studies have reported that pNGAL can detect early ischemia/reperfusion injury characterized by renal tubular injury in vivo [59,66] and distinguish AKI from CKD in dogs with azotemia [31]. Our results align with these studies and go beyond demonstrating elevated pNGAL levels even in stage 1 CKD—a stage that is difficult to distinguish based solely on SDMA or sCr levels. In contrast, a retrospective study examining the correlation between serum NGAL and several renal biomarkers, including histopathology, could not identify better candidates than existing methods, such as sCr and urinary protein concentrations [10]. Furthermore, several studies described serum NGAL as an insufficient marker, as compared with urinary NGAL, in dogs with experimental leishmaniasis [67], sepsis requiring emergency surgery [68], and parvovirus-derived AKI [40]. However, these studies reporting inferior pNGAL ability produced different results from ours, likely owing to limitations in the study population, such as a lack of comparison with normal subjects, a lack of consideration for breed and body weight, the absence of tubular lesions, few cases of renal disease confirmed by conventional methods, and a small sample size. Compared with previous studies, the present study confirmed the clinical and diagnostic value of pNGAL and showed significant differences in pNGAL concentrations by stage.

The other most-studied marker, KIM-1, is commonly used in histopathology to identify renal tubular injury [69]. However, the clinical relevance of urinary KIM-1 (uKIM-1) has been reported differently in the veterinary literature. A prospective study reported that uKIM-1 is useful in diagnosing early non-azotemic AKI [70]. In addition, uKIM-1 is useful for diagnosing envenomation-induced tubular injury [23], leptospirosis-induced tubular injury [71], babesiosis-induced tubular injury [72], and tenofovir disoproxil fumarate-induced mild kidney damage [57]. In contrast, several studies have reported that uKIM-1 is less reliable for detecting cisplatin-induced [73] and gentamycin-induced tubular injuries [53,56,74]. These differences are thought to occur because uKIM-1 appears during proliferation and regeneration, is related to fibrotic changes and interstitial damage, and is influenced by the detection time, method, and disease severity [56]. There is an abundance of literature on pNGAL; however, only a few studies have been conducted on pKIM-1 [1,22,48,75]. One study demonstrated that pKIM-1 could be an early detector of gentamicin-induced kidney injury in vivo [37]. To our knowledge, this is the first study to validate the clinical utility of pKIM-1 in veterinary medicine.

Despite the novelty of this study, it has several limitations related to its retrospective nature. Firstly, the age difference between the groups and possible subsequent effects could be overcome with a case collection method that minimizes bias, and a low correlation coefficient could be confirmed. Secondly, correlation analysis revealed that storage duration had a negligible effect on the outcomes of the biomarkers SDMA, pNGAL, and pKIM-1, with an association value of <0.05. This finding is consistent with that of previous research demonstrating the stability of NGAL in canine urine stored at -80 °C for at least 1 year [76] or even up to 8 years [60]. Various human studies have also validated the impact of storage conditions, duration, and freeze–thaw cycles on urinary NGAL and KIM-1 levels [77,78,79]. Compared with urine, plasma provides a neutral pH environment, offering better storage conditions for KIM-1 [80]; thus, the effect of storage duration on the results was minimal. However, it is important to acknowledge that the varying storage periods for each specimen may still be considered a limitation of this study. Third, owing to the limited number of tests, it was not feasible to consider other data, such as disease onset, prognosis, past concentration trends, urinalysis, GFR, and histopathology results, or the cause of renal injury and combinations of azotemia type. Furthermore, this study is restricted to small-breed dogs, which may limit the generalizability of the findings to other breeds or species. Therefore, the correlation results should be cautiously interpreted with the base condition, as the number of cases is small and a type II error may occur. Finally, the gold standard criteria should be applied to the ROC curve analysis; however, this study and several others in the literature used a clinical classification system (sCr) because the gold standard (i.e., GFR and histopathology) does not apply to routine practice. Despite these limitations, this study is noteworthy in confirming the significant differences in NGAL and KIM-1 plasma concentrations that are more applicable to veterinary practice according to the CKD stage at the clinical level. Together with existing biomarkers, these two biomarkers can contribute to the quantification of CKD risk and can be used for the diagnosis, treatment, monitoring, and management of CKD IRIS stage 1 in various diseases [1]. Nonetheless, future studies, including prospective, large-scale, long-term, and validation studies, are needed to overcome these limitations and apply these biomarkers in frontline clinics.

## 5. Conclusions

In conclusion, this study demonstrated that pNGAL and pKIM-1 concentrations could be used to discriminate a CKD risk group or stage 1 from CKD stages 2–4 in veterinary medicine. Therefore, this study lays the foundation for the future development of renal biomarkers for multiplex analysis.

## Figures and Tables

**Table 1:** The signalments and physical examination record.

Examination Record	Control (n = 11)		Risk (n = 14)		Stage 1 (n = 43)		Stage 2 (n = 33)		Stage 3–4 (n = 16)	
	Median (Q1–Q3)	N	Median (Q1–Q3)	N	Median (Q1–Q3)	N	Median (Q1–Q3)	N	Median (Q1–Q3)	N
Age (year)	6.0 (1.5–7.0)	11	10 * (8.1–11.8)	14	10.3 ** (9.0–12.0)	43	14.1 ***^,††,‡‡^ (12.0–15.3)	33	10.5 * (4.9–16.4)	16
NM(IM):NF(IF)	7(1):3(0)	11	11(0):2(1)	14	30(0):12(1)	43	17(2):13(1)	33	9(1):4(2)	16
BW (kg)	4.3 (3.2–4.9)	11	5.4 (4.6–6.5)	14	3.8 (3.2–4.8)	42	4.4 (3.0–5.4)	33	4.3 (3.3–6.5)	16
BCS (score)	4.3 (3.6–5.1)	10	4.9 (4.6–6.3)	13	3.8 (3.1–4.6)	40	4.6 (3.0–5.4)	32	4.5 (3.5–6.5)	15
sysBP (mmHg)	127.0 (114.5–136.0)	7	139.0 (126.0–144.0)	13	137.0 (121.5–145.8)	38	134.5 (126.3–141.8)	32	140.0 (131.5–155.0)	15

Median (interquartile range). The N column represents the number of recorded subjects for each object. NM, neutered male; IF, intact female; IM, intact male; NF, neutered female; BW, body weight; BCS, body condition score; sysBP, systolic blood pressure. sysBP < 140 can be classified as normotensive; 140–159, prehypertensive; 160–179, hypertensive; >180, severely hypertensive. Superscript * indicates a value in comparison to the control group; †, compared to the risk group; ‡, compared to the stage 1 group; * *p*-value < 0.05; **, ††, ‡‡ < 0.01; *** < 0.001.

**Table 2:** Complete blood count and chemistry results related to anemia and inflammation.

Parameters		Control (n = 11)		Risk (n = 14)		Stage 1 (n = 43)		Stage 2 (n = 33)		Stage 3–4 (n = 16)	
	RI	Median (Q1–Q3)	N	Median (Q1–Q3)	N	Median (Q1–Q3)	N	Median (Q1–Q3)	N	Median (Q1–Q3)	N
Anemia		0 (0%)		2 (6%)		7 (23%)		8 (25%)		9 (56%)	
RBC (10^12^/L)	5.65–8.87	7.12 (6.96–7.46)	11	7.63 (6.70–8.32)	13	7.19 (6.25–7.70)	42	6.75 (6.09–7.43)	32	5.99 *^,†,‡‡^ (4.90–7.13)	16
HCT (%)	37.3–61.7	49.8 (46.5–51.6)	11	49.4 (47.1–54.0)	13	46.7 (40.3–50.2)	42	42.2 (38.5–47.8)	32	35.0 ***^,†††,‡‡‡,§§^ (27.0–44.1)	16
HGB (g/dL)	13.1–20.5	17.3 (16.2–18.1)	11	16.7 (15.2–18.0)	13	16.3 (14.0–17.7)	42	14.7 (13.5–16.9)	32	12.6 ***^,††,‡‡‡,§^ (10.1–15.6)	16
RETIC (K/µL)	10–110	84.5 (71.1–95.8)	11	88.5 (53.4–100.6)	13	87.8 (57.2–115.6)	42	88.9 (27.9–101.6)	32	42.1 ^‡‡‡^ (17.1–65.1)	16
Inflammation		0 (0%)		1 (3%)		13 (42%)		9 (28%)		11 (69%)	
WBC ^#^ (10^9^/L)	5.05–16.7	10.15 (8.22–11.09)	11	9.19 (8.07–12.84)	13	9.71 (7.56–13.56)	42	11.02 (7.62–14.18)	32	11.52 (10.42–14.13)	16
NEU ^#^ (K/µL)	2.95–11.6	6.41 (4.98–7.10)	11	7.82 (5.69–8.68)	13	7.19 (5.05–9.99)	42	7.99 (5.04–11.01)	32	8.91 ** (7.47–11.68)	16
LYM ^#^ (K/µL)	1.05–5.1	3.07 (1.81–3.37)	11	2.05 (1.25–2.75)	13	2.10 (1.64–2.67)	42	2.07 (1.64–2.38)	32	1.75 (1.43–2.31)	16
MONO ^#^ (K/µL)	0.16–1.12	0.34 (0.29–0.43)	11	0.42 (0.38–0.71)	13	0.46 (0.33–0.71)	42	0.46 (0.35–0.65)	32	0.68 ** (0.49–1.24)	16
CRP ^#^ (mg/dL)	0.1–1	0.3 (0.2–0.3)	8	0.3 (0.3–0.3)	5	0.4 (0.3–0.7)	19	0.7 ** (0.5–1.3)	14	3.7 ***^,†††,‡‡‡,§§^ (1.9–9.2)	11

Median (interquartile range). The N column represents the number of tested subjects for each object. Hashes (#) indicate that the markers were analyzed after log_2_ transformation, as they showed a log-normal distribution. Superscript * indicates a value compared to the control group; †, compared to the risk group; ‡, compared to the stage 1 group; §, compared to the stage 2 group; *, †, § *p*-value < 0.05; **, ††, ‡‡, §§ < 0.01; ***, †††, ‡‡‡ < 0.001. RBC, red blood count; HCT, hematocrit; HGB, hemoglobin; RETIC, reticulocyte count; WBC, white blood count; NEU, neutrophil count; LYM, lymphocyte count; MONO, monocyte count; CRP, C-reactive protein.

**Table 3:** Complete blood chemistry, chemistry, and urinalysis results related to kidney disease.

Parameters		Control (n = 11)		Risk (n = 14)		Stage 1 (n = 43)		Stage 2 (n = 33)		Stage 3–4 (n = 16)	
	RI	Median (Q1–Q3)	N	Median (Q1–Q3)	N	Median (Q1–Q3)	N	Median (Q1–Q3)	N	Median (Q1–Q3)	N
BUN ^#^ (mg/dL)	7–27	15 (13–16)	11	17 (16–19)	14	17 (12–23)	43	32 **^,†,‡‡‡^ (18–47)	33	54 ***^,†††,‡‡‡,§§§^ (45–81)	16
sCr ^#^ (mg/dL)	0.5–1.8	0.8 (0.6–0.9)	11	0.9 (0.7–1.0)	14	0.7 (0.6–0.9)	43	1.4 ***^,†,‡‡‡^ (0.9–1.7)	33	3.0 ***^,†††,‡‡‡,§§§^ (2.0–3.7)	16
BCR ^#^	4–27	20 (16–26)	11	21 (17–21)	14	24 (16–31)	43	26 (14–33)	33	20 (13–32)	16
SDMA ^#^ (µg/dL)	0–14	9 (8–10)	9	11 (9–12)	14	12 ** (10–15)	43	21 ***^,†††,‡‡‡^ (18–27)	33	39 ***^,†††,‡‡‡,§§§^ (31–51)	16
pNGAL ^#^ (ng/mL)		1.9 (1.7–2.9)	10	2.7 (1.9–3.9)	14	5.7 ***^,††^ (3.6–8.7)	41	6.6 ***^,††^ (5.0–9.1)	32	23.0 ***^,†††,‡‡‡,§§§^ (13.9–29.3)	15
pKIM–1 ^#^ (ng/mL)		2.3 (2.1–2.8)	10	3.7 * (3.3–4.2)	14	3.0 (2.7–3.8)	43	5.2 ***^,†,‡‡‡^ (3.8–6.5)	33	8.5 ***^,†††,‡‡‡,§§§^ (6.1–12.3)	16
TP (g/dL)	5.2–8.2	6.6 (6.4–6.9)	11	7.0 (6.9–7.3)	12	6.9 (6.6–7.3)	41	6.9 (6.5–7.4)	26	7.1 (6.6–7.3)	14
Albumin ^#^ (g/dL)	2.3–4.0	3.4 (3.3–3.5)	11	3.3 (3.1–3.4)	12	3.2 (3.1–3.4)	41	3.1 (2.8–3.3)	26	3.0 **^,‡^ (2.8–3.2)	14
Globulin ^#^ (g/dL)	2.5–4.5	3.2 (3.1–3.3)	11	3.8 (3.6–4.0)	12	3.7 (3.4–3.9)	40	3.5 (3.3–4.2)	26	4.0 ** (3.7–4.4)	14
A/G	0.7–2.0	1.1 (1.0–1.1)	11	0.9 (0.8–0.9)	12	0.9 (0.8–0.9)	41	0.8 * (0.7–1.0)	26	0.8 *** (0.6–0.8)	14
ALT ^#^ (U/L)	10–125	57 (45–60)	11	61 (57–85)	11	60 (43–109)	41	74 (44–131)	26	29 (25–41)	13
AST ^#^ (U/L)	0–50	34 (31–42)	10	34 (33–44)	6	37 (29–58)	28	30 (23–47)	18	45 (34–91)	8
ALP ^#^ (U/L)	23–212	48 (34–110)	11	134 (52–199)	11	116 (77–295)	42	107 (44–295)	26	209 * (130–303)	14
Amylase ^#^ (U/L)	500–1500	483 (456–525)	4	522 (459–565)	4	633 (541–811)	20	725 (700–937)	10	1800 ***^,†††,‡‡‡,§§§^ (1579–3377)	7
Lipase ^#^ (U/L)	200–1800	835 (643–941)	4	1005 (792–1228)	6	682 (555–928)	20	652 (465–923)	11	976 ^§^ (827–4017)	7
CK ^#^ (U/L)	10–200	228 (77–233)	5	81 (57–116)	5	85 (53–123)	17	61 (59–92)	9	237 (169–318)	5
Osmolality (mOsm/kg)	290–330	306 (304–308)	8	305 (299–309)	10	304 (297–309)	31	307 (301–316)	25	315 ^†,‡‡‡,§§^ (311–330)	11
Na^+^ (mEq/L)	144–160	154 (153–156)	8	153 (151–156)	11	154 (151–157)	23	152 ^‡^ (147–154)	31	152 (147–154)	14
K^+ #^ (mEq/L)	3.5–5.8	4.2 (4.0–4.4)	8	4.1 (3.9–4.5)	11	4.1 (3.8–4.5)	23	4.7 (4.3–4.9)	31	4.9 (3.8–5.9)	14
Ca^2+^ (mg/dL)	7.9–12	9.7 (9.6–9.9)	4	10.1 (9.9–10.2)	6	9.8^†^ (9.4–10.3)	23	10.1 (9.4–10.7)	18	10.3 (9.8–10.8)	12
Cl^−^ (mmol/L)	109–122	118 (115–118)	8	114 (113–116)	11	114 (112–116)	22	112 (110–115)	31	111 (110–114)	14
PO^4– #^ (mg/dL)	2.5–6.8	2.7 (2.5–3.3)	4	3.2 (2.6–3.6)	8	4.0 (3.6–5.1)	23	4.3 (3.9–4.7)	20	8.2 ***^,†††,‡‡‡,§§§^ (5.8–11.5)	13
UP ^#^ (mg/dL)		4 (4–4)	1	29 (21–34)	4	46 (14–102)	24	30 (15–47)	18	57 (36–236)	11
UC ^#^ (mg/dL)		74 (74–74)	1	76 (72–85)	4	62 (17–127)	24	73 (54–95)	18	56 (23–64)	11
UPC ^#^	0–0.5	0.05 (0.05–0.05)	1	0.37 (0.28–0.42)	4	0.17 (0.09–1.48)	24	0.39 (0.16–0.96)	18	2.29 (1.01–5.86)	11
USG ^#^	1.001–1.030	1.016 (1.016–1.016)	1	1.028 (1.020–1.035)	2	1.027 (1.019–1.034)	28	1.014 (1.013–1.019)	16	1.011 ^†^ (1.008–1.014)	9

Median (interquartile range). The N column represents the number of tested subjects for each object. Hashes (#) indicate that the markers were analyzed after log_2_ transformation, as they showed a log-normal distribution. Superscript * indicates a value compared to the control group; †, compared to the risk group; ‡, compared to the stage 1 group; §, compared to the stage 2 group; *, †, ‡, § *p*-value < 0.05; **, ††, §§ < 0.01; ***, †††, ‡‡‡, §§§ < 0.001. BUN, blood urea nitrogen; sCr, serum creatinine; BCR, blood urea nitrogen-to-creatinine ratio; SDMA, symmetric dimethylarginine; pNGAL, plasma neutrophil gelatinase-associated lipocalin; pKIM-1, plasma kidney injury molecule-1; A/G, albumin-to-globulin ratio; ALT, alanine aminotransferase; AST, aspartate aminotransferase; ALP, alkaline phosphatase; CK, creatinine kinase; TP, total protein; UP, urine protein; UC, urine creatinine; UPC, urine protein creatinine ratio; USG, urine-specific gravity.

**Table 4:** Renal risk-detecting cut-off values for determining the novel upper limit in International Renal Interest Society stages 1–4, including kidney injury risk.

Parameters	Cut-Off (Unit)	AUC (95% CI)	Sensitivity, % (95% CI)	Specificity, % (95% CI)	LR^+^	LR^−^
sCr	0.95 (mg/dL)	0.71 * (0.59–0.82)	50.94 (41.56–60.26)	90.91 (62.26–99.53)	5.60	0.54
SDMA	10.50 (µg/dL)	0.86 *** (0.76–0.95)	83.02 (74.75–88.98)	77.78 (45.26–96.05)	3.74	0.22
pNGAL	3.30 (ng/mL)	0.90 *** (0.83–0.97)	81.37 (72.73–87.74)	90.00 (59.58–99.49)	8.14	0.21
pKIM-1	3.32 (ng/mL)	0.90 *** (0.82–0.99)	66.04 (56.60–74.35)	100 (72.25–100.00)	-	0.34

Abbreviations: AUC, area under the curve; CI, confidence interval; LR^+^, positive likelihood ratio; LR^−^, negative likelihood ratio; pNGAL, plasma neutrophil gelatinase-associated lipocalin; pKIM-1, plasma kidney injury molecule-1; SDMA, symmetrical dimethylarginine; sCr, serum creatinine. * *p* < 0.05, *** *p* < 0.001.

**Table 5:** CKD stage 1-classifying cut-off values for determining the novel upper limit in International Renal Interest Society stages 1–4.

Parameters	Cut-Off (Unit)	AUC (95% CI)	Sensitivity, % (95% CI)	Specificity, % (95% CI)	LR^+^	LR^−^
sCr	0.95 (mg/dL)	0.65 * (0.55–0.75)	53.26 (43.14–63.12)	76.00 (56.57–88.50)	2.22	0.62
SDMA	13.50 (µg/dL)	0.84 ** (0.77–0.92)	70.65 (60.67–78.98)	91.30 (73.20–98.45)	8.12	0.32
pNGAL	4.19 (ng/mL)	0.88 *** (0.80–0.95)	76.14 (66.26–83.83)	87.50 (69.00–95.66)	6.09	0.27
pKIM-1	3.70 (ng/mL)	0.72 *** (0.62–0.82)	57.61 (47.41–67.20)	79.17 (59.53–90.76)	2.77	0.54

Abbreviations: AUC, area under the curve; CI, confidence interval; LR^+^, positive likelihood ratio; LR^−^, negative likelihood ratio; pNGAL, plasma neutrophil gelatinase-associated lipocalin; pKIM-1, plasma kidney injury molecule-1; SDMA, symmetrical dimethylarginine; sCr, serum creatinine. * *p* < 0.05, ** *p* < 0.01, *** *p* < 0.001.

**Table 6:** Conventional CKD classifying cut-off values to determine the traditional upper limit in International Renal Interest Society stages 2–4.

Parameters	Cut-Off (Unit)	AUC (95% CI)	Sensitivity, % (95% CI)	Specificity, % (95% CI)	LR^+^	LR^−^
sCr	1.25 (mg/dL)	0.89 *** (0.83–0.96)	69.39 (55.47–80.48)	95.59 (87.81–98.80)	15.73	0.32
SDMA	16.50 (µg/dL)	0.95 *** (0.90–0.99)	85.71 (73.33–92.90)	98.48 (91.90–99.92)	56.39	0.15
pNGAL	4.89 (ng/mL)	0.75 *** (0.66–0.84)	82.98 (69.86–91.11)	60.00 (47.86–71.03)	2.07	0.28
pKIM-1	4.14 (ng/mL)	0.88 *** (0.81–0.95)	79.59 (66.36–88.52)	86.57 (76.40–92.77)	5.93	0.24

According to IRIS 2019, 18< SDMA (µg/dL) < 35 or 1.4 < sCr (mg/dL) < 2.8 indicates a mild increase (CKD stage 2), 36 < SDMA < 54 or 2.9 < sCr < 5.0 indicates a moderate increase (CKD stage 3), whereas 54 < SDMA or 5.0 < sCr indicates an increasing risk of systemic signs and uremic crises. Abbreviations: AUC, area under the curve; CI, confidence interval; IRIS, International Renal Interest Society; LR^+^, positive likelihood ratio; LR^−^, negative likelihood ratio; pNGAL, plasma neutrophil gelatinase-associated lipocalin; pKIM-1, plasma kidney injury molecule-1. ***p < 0.001.

## Data Availability

The datasets used and/or analyzed for this study are available from the corresponding author upon reasonable request.

## Supplementary Materials

The following supporting information can be downloaded at: https://www.mdpi.com/article/10.3390/ani14162313/s1, Figure S1: Correlation between traditional and novel kidney injury biomarkers with linear interval axis presentation.; Figure S2: Normal and log-normal Q-Q plots of each dataset.; Figure S3: Statistical analysis of concentration differences in biomarkers between groups before log transformation.
