# Supplementary figures and images for: Early Detection of Chronic Kidney Disease Using Plasma Neutrophil Gelatinase-Associated Lipocalin and Kidney Injury Molecule-1 in Small-Breed Dogs: A Retrospective Pilot Study
Source: Animals (Basel). 2024 Aug 9;14(16):2313. doi: 10.3390/ani14162313 (PMC11350678; doi:10.3390/ani14162313)

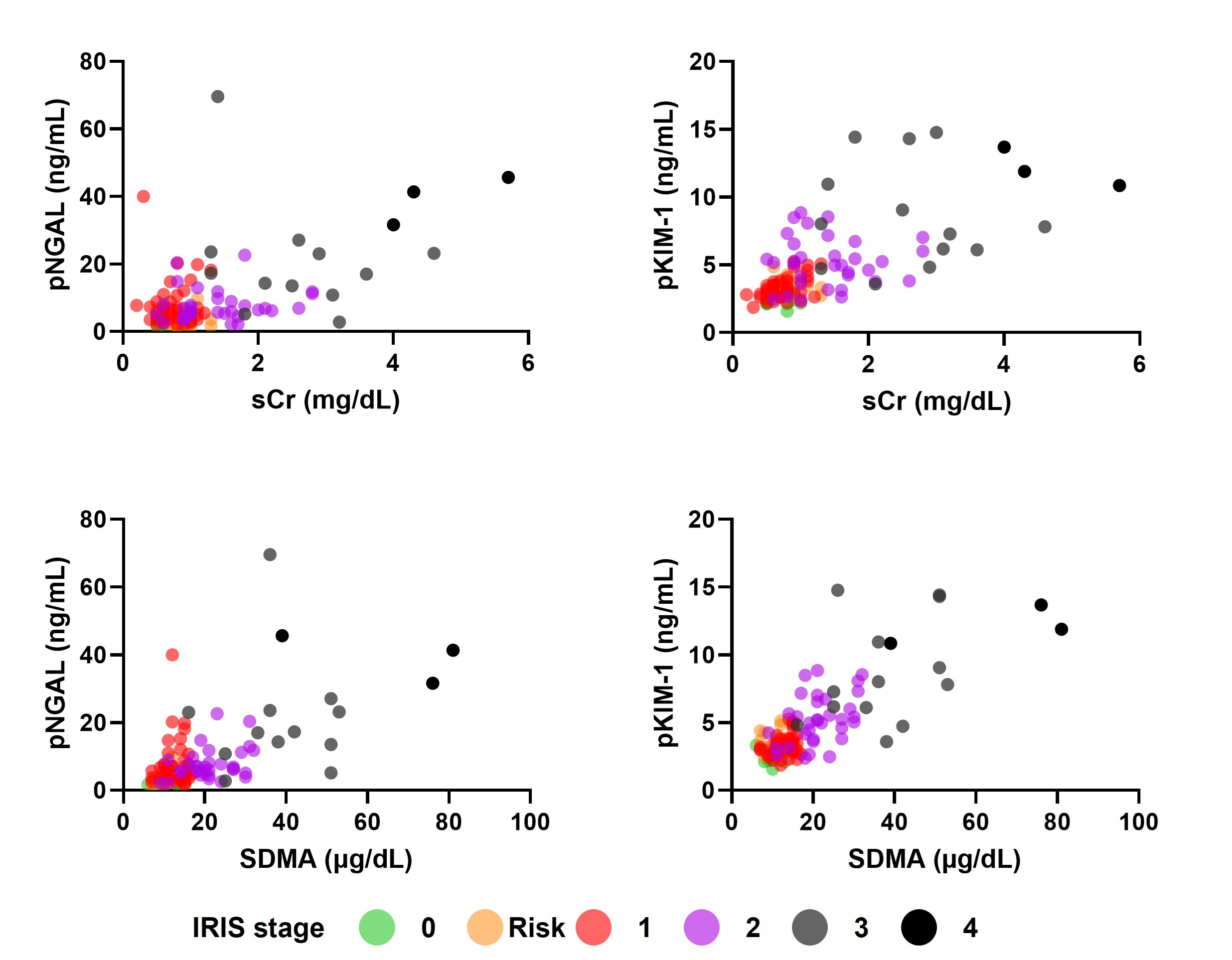

Supplement: Supplementary file 1 [file animals-14-02313-s001.zip › Supplementary Figure 1.jpg]

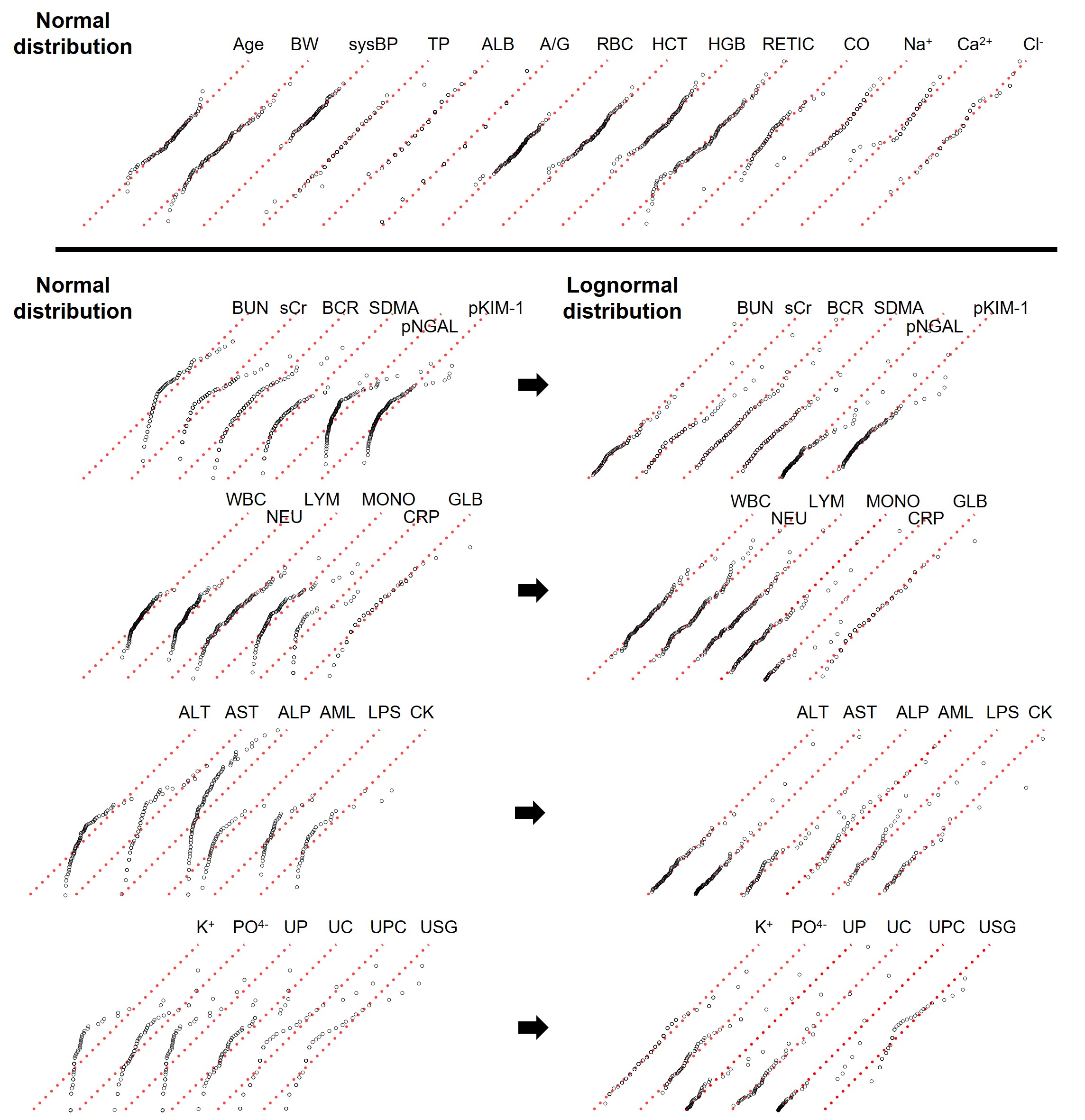

Supplement: Supplementary file 1 [file animals-14-02313-s001.zip › Supplementary Figure 2.jpg]

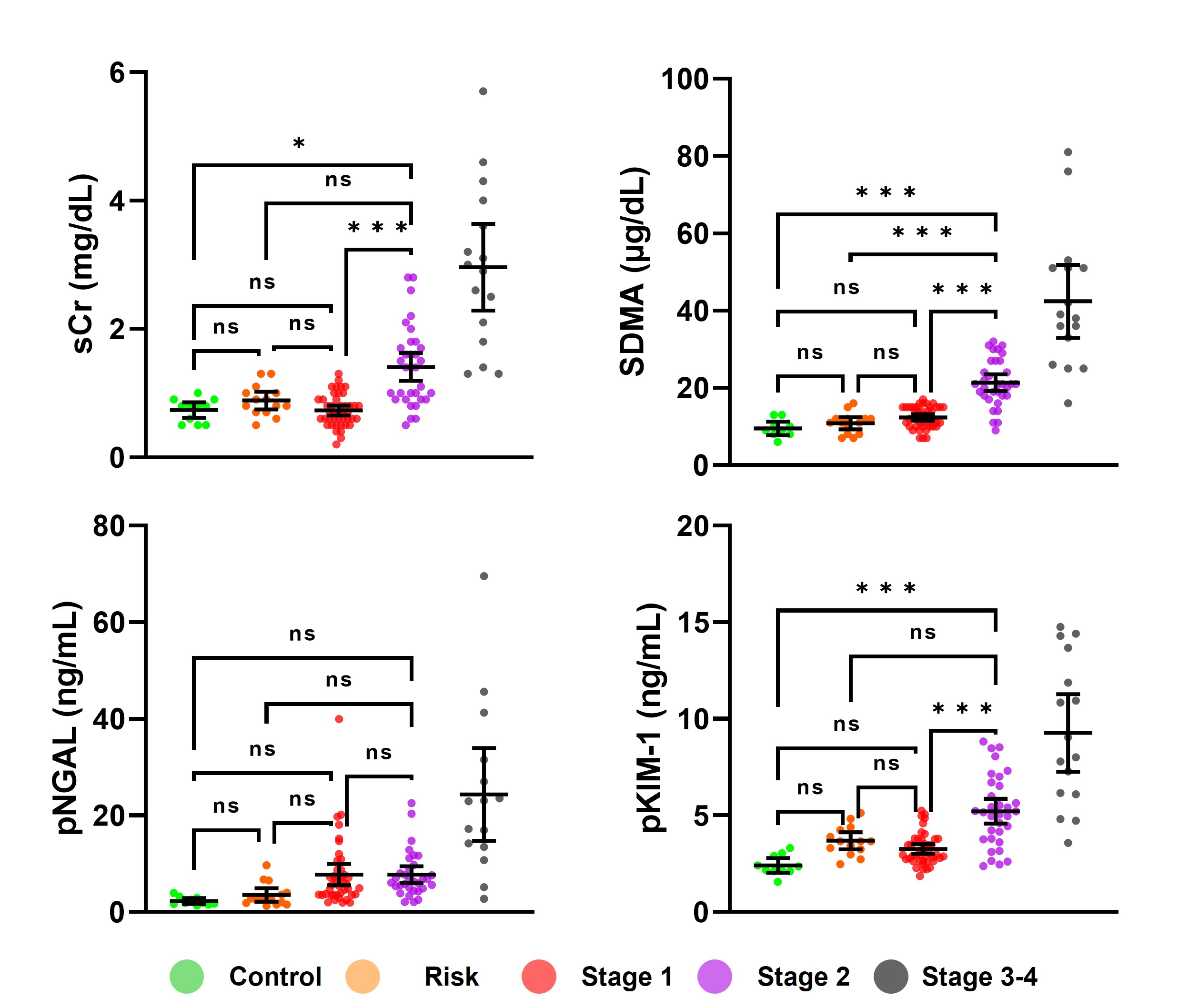

Supplement: Supplementary file 1 [file animals-14-02313-s001.zip › Supplementary Figure 3.jpg]
